# JOINT ASSOCIATION OF VISIBLE AND SELF-PERCEIVED AGING WITH PREMATURE MORTALITY

**DOI:** 10.1093/geroni/igae098.3061

**Published:** 2024-12-31

**Authors:** Minghao Kou, Hao Ma, Xuan Wang, Lu Qi

**Affiliations:** Tulane University, New Orleans, Louisiana, United States; Tulane University, New Orleans, Louisiana, United States; Tulane University, New Orleans, Louisiana, United States; Tulane University, New Orleans, Louisiana, United States

## Abstract

This study quantifies the association of a combination of visible and self-perceived aging indicators with the risk of all-cause and cause-specific premature mortality. Utilizing data from 369,741 UK Biobank participants initially free of cardiovascular disease and cancer, with follow-up until December 31, 2022, we analyzed four aging indicators: hearing loss, teeth loss, self-perceived age, and falls. Using Cox proportional hazards models, we assessed their joint association with mortality outcomes over a median follow-up of 13.74 years, during which 22,934 cases of premature mortality were documented. Compared to participants without aging indicators, those exhibiting all indicators experienced a 81% (95% confidence interval, 59%–107%), 96% (47%–160%), 55% (26%–91%), and 114% (73%-165%) higher risk of all-cause, cardiovascular disease, cancer, and other causes of premature mortality, respectively. Notably, these associations were more pronounced in younger participants, those with unhealthy lifestyles, and lower socioeconomic status (P < 0.05 for interactions). Additionally, we identified a significant additive interaction between aging indicators and frailty index, contributing an additional 16.08% (95%CI, 7.91-24.25%) risk of premature mortality. In summary, the combination of visible and self-perceived aging indicators was associated with a higher risk of all-cause and cause-specific premature mortality. These findings highlight the importance of considering both visible and self-perceived aging indicators in health risk assessments and intervention planning.

